# Study of resistance mechanism of Alternaria blight (*Alternaria brassicicola*) by biochemical markers in Indian Mustard (*Brassica juncea* L. Czern. &Coss.)

**DOI:** 10.3389/fpls.2024.1420197

**Published:** 2024-08-15

**Authors:** Anurag Mishra, Nawaz Ahmad Khan, Ratnesh Kumar Jha, Tamilarasi Murugesh, Ashutosh Singh

**Affiliations:** ^1^ Department of Plant Molecular Biology and Genetic Engineering, Acharya Narendra Deva University of Agriculture and Technology, Ayodhya, Uttar Pradesh, India; ^2^ Department of Agricultural Biotechnology and Molecular Biology, Dr. Rajendra Prasad Central Agricultural University, Samastipur, Bihar, India; ^3^ Centre for Advanced Studies on Climate Change, Dr. Rajendra Prasad Central Agricultural University, Samastipur, Bihar, India

**Keywords:** mustard, Alternaria disease, biochemical, catalase, sulfur-containing amino acids

## Abstract

Indian mustard (*Brassica juncea*) is an important oilseed crop in India. Alternaria leaf spot (Alternaria blight) is incited by the fungus *Alternaria brassicicola*. It majorly affects crop production leading to a yield loss of up to 70%. To circumvent this problem, the study of the resistance mechanism and identification of biochemical markers is one of the important strategies for its management. In the present study, a total of 219 genotypes of Indian mustard with check were screened for Alternaria blight over two seasons. Based on the area under the disease progress curve (AUDPC) scores, ten consistently performing genotypes were selected for the screening of biochemical and yield attributes under artificial inoculated conditions of *Alternaria brassicicola (Berk) Sacc.* The result showed a negative correlation between disease and yield attributes. The catalase (CAT) activity was significantly increased in resistant genotypes compared to susceptible ones, indicating the crucial role of CAT in the resistance mechanism. Pathogen infection also increases the total protein content and the Alternaria-resistant genotype showed the highest total soluble protein while the susceptible genotype showed the lowest total soluble protein. The ten genotypes were categorized by SSI (stress susceptibility index) and Varuna was identified as a tolerant genotype and Giriraj as a susceptible genotype for *Alternaria brassicicola* (Berk) Sacc. Varuna and Giriraj were chosen for quantitative analysis of methionine and tryptophan amino acids from seeds using RP-HPLC (Reverse Phase-High Performance Liquid Chromatography) and there were significant differences in the levels of methionine and tryptophan between the Varuna and Giriraj genotypes. Varuna showed higher methionine and tryptophan content compared to the Giriraj genotype. Higher protein content demonstrated an increase in biotic stress-responsive amino acids, such as methionine and tryptophan, suggesting increased resistance to Alternaria diseases in these high-protein genotypes. These amino acids could be used as biochemical markers for Alternaria resistance of mustard.

## 1 Introduction

Oilseed crops play a key role in the Indian agricultural economy and account for 19% of global acreage but contribute only 2.7% to global production. India is the third largest consumer and importer of edible oils and mainly relies on imports. India imports approximately $10 billion in edible oil annually (Reddy and Immanuelraj, 2017; Jha, 2019; Singh et al., 2021a; Khan et al., 2023). Mustard is commercially significant and it is most widely cultivated due to its resilient nature across diverse agroclimatic conditions (Singh et al., 2018, Singh et al., 2020a, b). Mustard productivity in India is 1.2 t/ha while in Germany productivity is 3.73t/ha (Tyagi and Singh, 2016). Indian mustard confronts a range of challenges, including biotic and abiotic stresses, that limit its productivity (Singh et al., 2021b).

Among the biotic stresses, *Alternaria brassicicola (Berk) Sacc*, (Saccardo, 1886) caused severe yield loss (up to 70%) (Gupta et al., 2020). *Alternaria brassicicola* infections cause dark brown lesions on leaves, stems, and siliquae, which, in turn, diminish photosynthetic efficiency, hasten senescence, and ultimately result in crop losses (Hansen and Earle, 1997; Nowakowska et al., 2019). This disease may cause significant loss in both temperate and tropical regions (Mathpal et al., 2016). In India, *A. brassicicola* reduces yield by up to 47% (Sharma et al., 2013). Initial symptoms of Alternaria produce a series of concentric rings (Mamgain et al., 2013). It is a necrotrophic pathogen that causes lesions in leaves, stems, and siliquae that significantly affect the quality and quantity of mustard seeds by reducing oil content, seed size, and seed color (Duczek et al., 1999). It is important to gain insight into genotypic variability in *Alternaria brassicicola* resistance among mustard crops as it could be used as a potential donor for the development of a resistant variety. Phenotyping over multiple seasons provides stable performance of genotypes (Li and Wu, 2023).

Plant cellular antioxidant enzyme activity is a biochemical response to disease stress. It is considered useful for the early detection of disease and is also associated with Alternaria resistance in mustard. Moreover, successful infections disrupt cell wall proteins and trigger an overproduction of reactive oxygen species (ROS) (Meena et al., 2016a; Narware et al., 2023). ROS plays a critical role in plant development and defense but is often linked to disease susceptibility (Bandyopadhyay et al., 1999). Excessive ROS production can lead to cell membrane damage, protein degradation, and harm to the photosynthetic machinery, causing oxidative stress in plants (Das and Roychoudhury, 2014). To counteract these effects, plants rely on antioxidants, which act as scavengers (Shereefa and Kumaraswamy, 2016). The susceptibility of plants to necrotrophic fungi, such as *Alternaria brassicicola (Berk) Sacc.*, is closely tied to the balance between ROS generation and scavenging through antioxidant defense mechanisms (Sharma et al., 2012). An imbalance in this process indicates a failure of host defense or successful infection. Peroxidase is a ROS scavenger that converts hydrogen peroxide (H_2_O_2_) to water and plays a critical role in preventing oxidative damage and has been implicated in various defense-related processes, including hypersensitive response, lignification, cross-linking of phenolics and glycoproteins, suberization, and phytoalexin production (Jung et al., 2004; Kashyap et al., 2023). Additionally, catalase is frequently employed by cells to rapidly break down hydrogen peroxide into less reactive gaseous oxygen and water molecules, thereby preventing cellular disintegration (Bolwell and Wojtaszek, 1997; Kannojia et al., 2017). These mechanisms represent the most common means of scavenging ROS during stress responses and play a significant role in plant resistance (Mittler, 2002; Kesharwani et al., 2023).

In this context, sulfur (S) emerges as a vital macronutrient for plants, playing a crucial role in fundamental plant processes and the regulation of various metabolic pathways (Rathore et al., 2015; Hasanuzzaman et al., 2018). Additionally, sulfur plays a critical role in shielding plants from adverse environmental conditions. Sulfur-containing amino acids and metabolites are instrumental in maintaining plant cell mechanisms, thereby enhancing their capacity to withstand Alternaria stress (Hasanuzzaman et al., 2018). Furthermore, sulfur and its derivatives, including glutathione (GSH), hydrogen sulfide (H_2_S), methionine (Met), cysteine (Cys), phytochelatin (PC), ATP sulfurylase (ATPS), protein thiols, and others have been observed to fortify antioxidant defenses and mitigate the excessive production of ROS under various biotic stress conditions (Capaldi et al., 2015). Notably, plants from the Brassicaceae family, including important crops, exhibit a higher demand for sulfur compared to other plant varieties to achieve optimal growth and yield. Sulfur is primarily stored in the form of storage proteins, such as cruciferin and napin, with sulfur-rich secondary metabolite referred to as glucosinolate (GSL) (Borpatragohain et al., 2019).

With the background of the above points, this study aimed to screen large populations of Indian rapeseed mustard for Alternaria disease over two seasons to identify sources of disease resistance within rapeseed and field mustard germplasm. Further biochemical analysis and morphological markers studies were conducted to elucidate resistance mechanisms against the Brassica species.

## 2 Materials and methods

### 2.1 Material

A total of 219 genetically diverse *Brassica juncea* genotypes with a check genotype (Giriraj) were collected from the Department of Genetics and Plant Breeding, NDUAT Faizabad, Uttar Pradesh India, and the Directorate of Rapeseed-Mustard Research, Bharatpur, Rajasthan, India.

### 2.2 Screening of mustard genotypes for Alternaria blight

A total of 219 genotypes with susceptible check Giriraj (Dhaliwal and Singh, 2019) of *Brassica juncea* were sown for evaluation of *Alternaria brassicicola* during two crop seasons (2016-17 and 2017-18) under natural field conditions. They were grown in augmented block design. The check was sown after every ten genotypes. Each genotype was sown in two rows with a row length of 1.5 m and a spacing of 30x10 cm. All standard agronomic practices were followed to raise the crops. The genotypes were meticulously assessed and categorized based on their *Alternaria blight* disease severity scores at the reproductive stage. The disease severity scores of *Alternaria blight* are presented in **Table 1** (AICRP and Proceeding, 2011).

**Table 1:** Details of the rating scale and AUDPC (area under the disease progress curve) range used for rating Alternaria blight in rapeseed mustard.

S. No.	Rating Scale (0-9)	Description of scale	AUDPC range	Host reaction
1	0	No visible symptoms	0	Near Immune (I)
2	1	< 5% leaf area covered	≤ 50	Resistant (R)
3	3	5-10% leaf or pod area covered with small pinhead spots on the leaves and superficial pinhead spots on pods	51 - 100	Moderate Resistant (MR)
4	5	11-25% leaf or pod area covered with small spots on leaf and superficial pinhead spots on pods	101-250	Moderately Susceptible (MS)
5	7	26-50% leaf or pod area covered with bigger spots with the initiation of coalesces on leaves and deep lesions on pods	251-500	Susceptible (S)
6	9	> 50% leaf or pod area covered with bigger commonly coalescing spots on leaves and deep lesions on pods	≥ 500	Highly Susceptible (HS)

#### 2.2.1 Measurement of disease severity

To evaluate disease severity, five plants were randomly selected for each genotype and tagged. Infection levels and disease appearance were monitored, and severity was visually recorded for the tagged plants using a rating scale ranging from 0 to 9. It was recorded at three crop growth stages: 60 DAS, 75 DAS, and 90 DAS in both crop seasons (2016-17 and 2017-18). To quantify the disease progress, the area under the disease progress curve (AUDPC) was calculated using the midpoint rule method (Campbell and Madden, 1990). The details of the formula are as follows:


$$\text{AUDPC }=\sum_{\text{i}=1}^{\text{n}-1}\left[\left({\text{t}}_{\text{i}+1}–{\text{ t}}_{\text{i}}\right)\;\left({\text{y}}_{\text{i}}+{\text{ y}}_{\text{i}+1}\right)/2\right]$$

Where,

y= Percentage of affected foliage at each reading.
t = Time in days between each reading.
n = Total number of readings.

#### 2.2.2 Isolation of *Alternaria brassicicola* and inoculum preparation

*Alternaria brassicicola* was isolated from leaf samples of infected plants for artificial inoculation in the treated block. The leaf samples were sterilized in 0.5% sodium hypochlorite solution for 1-2 minutes. They were subsequently rinsed thoroughly with distilled water and placed on potato dextrose agar (PDA) culture media (G-Biosciences Geno Technology USA). The cultures were incubated at a temperature of 27°C and kept in 12-hour cycles of light and darkness for 6-10 days for fungal growth. *Alternaria brassicicola* was confirmed by microscopic examination. The morphological characteristics of *Alternaria brassicicola (Berk) Sacc.* such as conidia shape, structure, and size were observed under the microscope (Meena et al., 2016b). A *Alternaria brassicicola* colony was introduced into potato dextrose broth (PDB) and incubated for 10 days. During the incubation periods, the temperature was maintained at 27°C and followed by 12 hours of daylight and 12 hours of darkness. Further, the concentration of the inoculum was carefully determined using a hemocytometer (Avni Scientific Co.) with a size of 30 x 70 mm and 4 mm thickness and adjusted to a level of 5 x 10^^4^/mL (Akhtar et al., 2007).

#### 2.2.3 Evaluation of selected genotypes for morphological and yield traits

A total of 10 sets of consistently performing genotypes were identified after a 2-year field screening which included moderate resistant (MR) (2), moderate susceptible (MS) (1), susceptible (S) (3) and highly susceptible (HS) (4). . These 10 genotypes were sown during the 2018-19 rabi season at the Agricultural Research Farm, NDUAT, Faizabad, India. The experiment was conducted in two blocks in natural field conditions with three replications and a spacing of 30 x 10 cm. Isolated spores of *Alternaria brassicicola* were sprayed (75 DAS) in one block and it was considered a treated block (Chakrabarty et al., 2018). The others that had not been sprayed with spores of *Alternaria brassicicola* were considered as control. In the control block, 0.02% mancozeb fungicide solutions (non-inoculated) were sprayed to control Alternaria blight.

#### 2.2.4 Data collection of genotypes for morphological and yield attributes

Five plants were randomly tagged from each genotype in each replication to record the observations. The following data were recorded from three replications.

**Plant height (PH):** At the time of maturity, plant height was measured from the ground to the main shoot tip.

**Number of pods per plant (PB):** The total number of pods counted from each plant and considered as the number of pods per plant.

**Secondary branch (SB):** The number of secondary branches per plant was assessed by enumerating branches arising from primary branches.

**Mean raceme length (SMR) (cm):** The length of the main raceme was measured from the joint at the apex of the primary branch to the top of the plant, and the average was taken to represent the overall length.

**Siliqua length (SL) (cm):** It was measured by randomly selected siliquae from base to tip in centimeters.

**Seeds per siliqua (SPS):** The number of seeds per siliquae was calculated by counting from five randomly chosen siliquae in each of the five tagged plants and the average value taken.

**Siliquae per plant (SPP):** the mean of the total number of siliquae counted from the main raceme.

**Test weight (TW) (g):** 1,000 sun-dried seeds from each selected plant were counted and weighed in grams using an electronic balance.

**Seed yield per plant (YPP):** The seed yield per plant (g) was recorded by weighing the total seeds obtained after threshing each plant separately.

**Area under the disease progress curve (AUDPC):** The calculation of the area under the disease progress curve (AUDPC) was based on severity scores during the reproductive stage.

#### 2.2.5 Biochemical analysis

##### 2.2.5.1 Estimation of total soluble proteins

Samples were collected from all 10 genotypes in both the non-inoculated and inoculated conditions 7 days after fungal inoculation on the leaves (82 DAS). A total of nine plants from each genotype were selected. The leaf samples were promptly frozen and stored at a temperature of -80°C. The total soluble protein (TSP) of the leaves was quantified by using methods of Bradford, 1976; Sambrook and Russell, 2001; and Kruger, 2009.

##### 2.2.5.2 Peroxidase activity

Peroxidase activity was estimated from control and treated plants. A fresh leaf sample (200g) was homogenized in 10 ml of phosphate buffer (pH 6.0) and then centrifuged at 10,000 rpm for 30 min at room temperature. Afterward, 2 ml of the enzyme extract was mixed in a test tube containing 2 ml of phosphate buffer (pH 6.0) with 1 ml of pyrogallol and 0.2 ml of H_2_O_2_. The mixture was incubated at 37°C, shaken, and placed in water for 10 minutes to allow purpurogallin formation. The color intensity was measured at 430 nm using a spectrophotometer to assess the enzyme’s activity (McCune and Galston, 1959).

##### 2.2.5.3 Catalase activity

The catalase activity was assessed by Dhindsa et al. (1981) method. The reaction mixture, with a final volume of 1 ml, was prepared and contained 50 mM sodium phosphate buffer (pH 7.0) with 50 µl of enzyme extract. To determine the enzyme activity, 35 µl of H_2_O_2_ was added at every 5-second interval over a duration of 1 minute. The rate of decreasing absorbance at 240 nm was measured. The catalase activity was quantified by using an extinction coefficient of 39.4 M^-1^ cm^-1^.

#### 2.2.6 Stress Susceptibility Index (SSI)

The SSI was calculated for all morphological, physiological, and biochemical traits to categorize the genotypes, and it was calculated by using the following formula:


SSI=(1−Y/Yp)/(1−X/Xp)

Where:

Y represents the mean performance of a mustard genotype in an inoculated condition.
Yp represents the mean performance of a mustard genotype in a non-inoculated condition.
X represents the mean performance of all mustard genotypes in an inoculated condition.
Xp represents the mean performance of all mustard genotypes in a non-inoculated condition.

For each trait, genotypes with SSI values below 0.5 were categorized as resistant, those with values between 0.5 and 1 were considered moderately resistant, and genotypes showed values ≥1 were considered as susceptible genotypes (Singh et al., 2024).

#### 2.2.7 Quantitative estimation of methionine and tryptophan amino acids by RP-HPLC

##### 2.2.7.1 Extraction of amino acids from *B. juncea*

The following procedure was used for methionine and tryptophan amino acids estimation.

##### 2.2.7.2 Preparation of extract

The SSI was used to identify two contrasting genotypes (Varuna and Giriraj) for Alternaria blight. These two contrasting genotypes were used for methionine and tryptophan amino acid profiling. The seeds from the genotypes were first stored in a deep freezer overnight. After 24 h, the seeds were finely ground into a powder using a mortar and pestle, and it was kept for vacuum drying.

The vacuum-dried samples were subjected to vapor-phase hydrolysis, using 200 µL of constantly boiling 6N HCl and 40 µL of phenol. Subsequently, the samples were oven-dried at a temperature range of 112-116°C for a duration of 20-24 hours to eliminate any excess HCl. Afterward, the tubes containing the samples were passed through a 90-min vacuum treatment. The seed samples were reconstituted by adding 500 µL of 20 mM boiling HCl.

##### 2.2.7.3 Derivatization of amino acids

Amino acids were derivatized (FMOC–AA) at room temperature using a Precolumn derivatization technique. Initially, a 300 µL aliquot of the mustard seed extract (or a standard amino acid solution) was combined with 600 µL of a 200 mM borate buffer (pH 10.0). Afterward, 600 µL of 15 mM FMOC chloride (in acetonitrile) was added to the mustard seed extract, initiating the derivatization process. The reaction was stopped after 5 min by introducing 600 µL of 300 mM ADAM (a mixture of water and acetonitrile in a 1:1 ratio) and it formed the FMOC–ADAM complex during a 1 min reaction. The sample was filtered and subjected to analysis using RP-HPLC. The entire procedure was completed within 6 minutes. The detection of amino acids was carried out using automated derivatization with FMOC–AA and online analysis was conducted using RP-HPLC with ultraviolet−visible (UV−Vis) detection (Henderson et al., 2003).

Filtered samples were analyzed using a HiQ Sil C18-HS column (4.6 mm × 250 mm × 5µm) and a Systronics high-performance liquid chromatography system equipped with a UV−Vis detector and autosampler. The analysis was monitored at a wavelength of 263 nm, and the column temperature was maintained at 25°C. The mobile phase consisted of an isocratic mixture prepared from 50 mM acetate buffer and acetonitrile (in a ratio of 70:30). The flow rate was set at 0.750 mL/min, and the injection volume was 20 μL. The total runtime for a single sample analysis was 25 min. Quantification of the various compounds was based on peak areas and expressed as equivalents of representative standard compounds. The results were expressed in grams per 100 g of fresh weight.

#### 2.2.8 Statistical analysis

Microsoft Excel 2016 Analysis Tools were utilized for a two-way ANOVA. R Studio was employed to explore Pearson’s correlation coefficient, conduct principal component analysis (PCA), and use Duncan’s multiple range test (DMRT) to categorize genotypes into different groups.

## 3 Results

### 3.1 Screening for Alternaria blight resistance: a comparative analysis of 2016-17 and 2017-18

**Figure 1:**
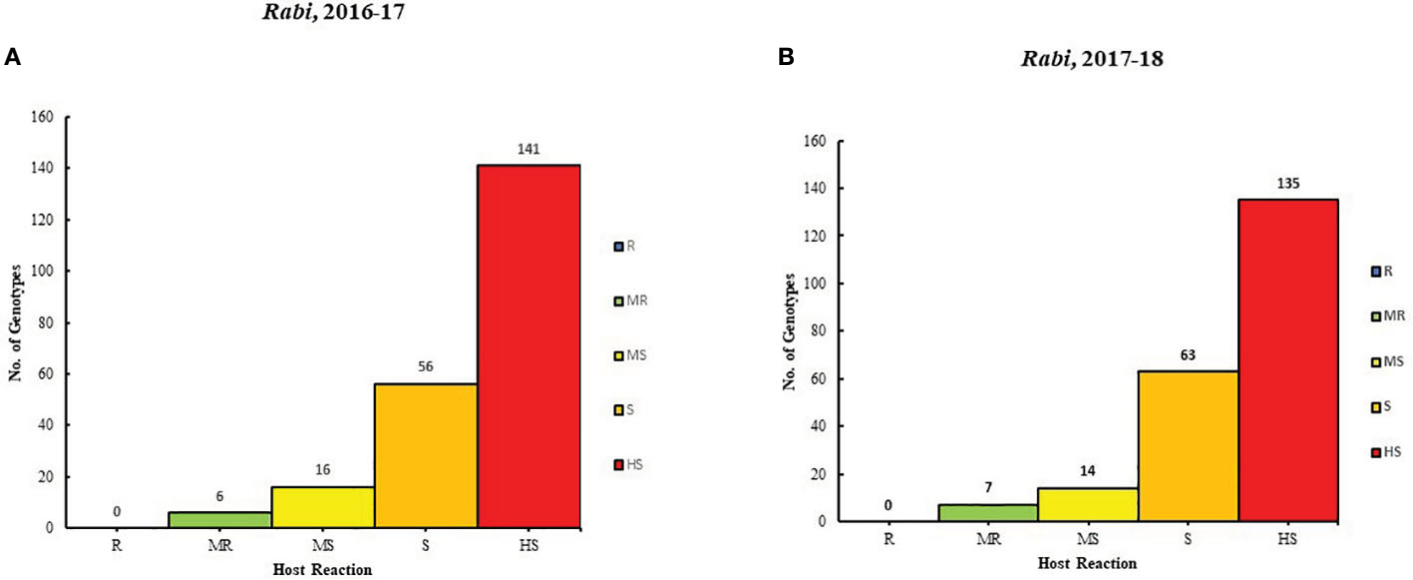
Genotype distribution in five host reaction categories to Alternaria blight during the rabi season for **(A)** 2016-17 and **(B)** 2017-18 under natural conditions.

The result of 2 years (2016-17 and 2017-18) of rigorous screening processes revealed varying degrees of resistance among different genotypes to Alternaria blight. Based on the AUDPC scores, six genotypes were categorized as moderately resistant (MR), 116 genotypes were moderately susceptible (MS), 56 were susceptible (S) genotypes, and 141 genotypes were highly susceptible (HS) in the year 2016-2017. Similarly, in 2017-18, the genotypes were classified: 7 genotypes were MR, 14 were MS, 63 were S, and 135 were HS. It is important to note that none of the genotypes showed resistance in both years (**Supplementary Table 1**; **Figures 1A, B**).

**Figure 2:**
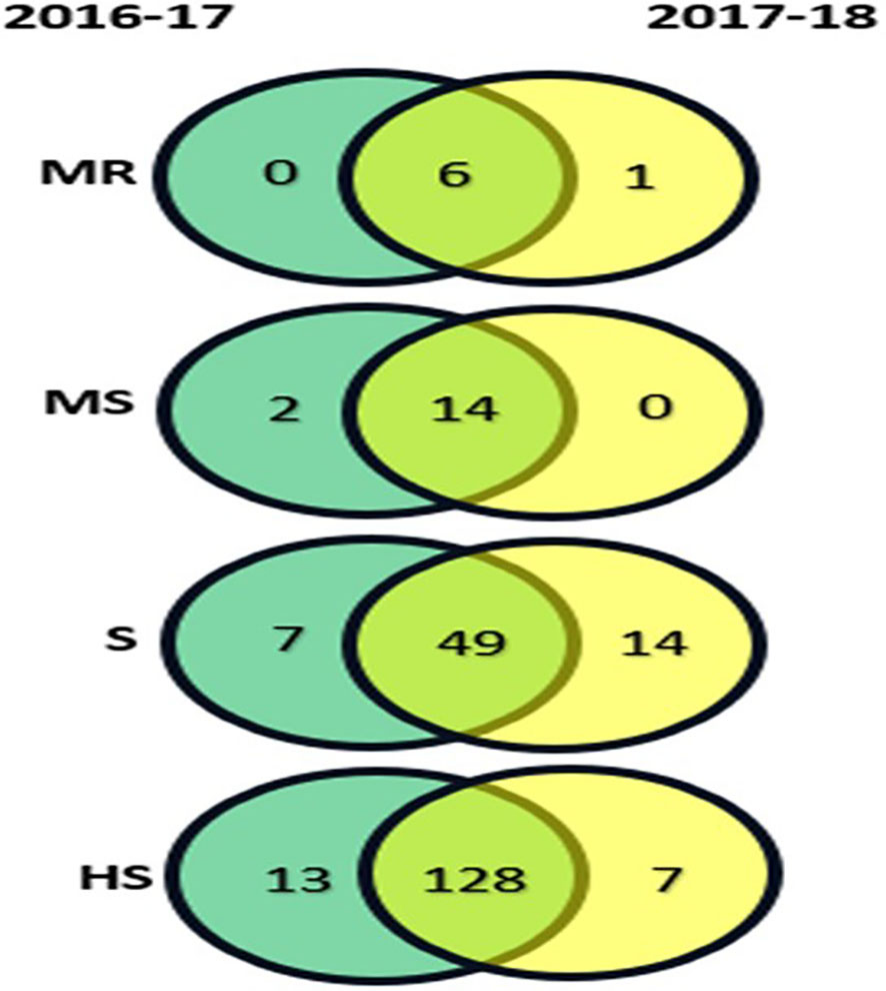
Host reaction categories for 219 mustard genotypes under natural field conditions during the rabi seasons of 2016-17 and 2017-18, with categories represented as : R, Resistant; MR, Moderately Resistant; MS, Moderately Susceptible; S, Susceptible; HS, Highly Susceptible.

A total of 10 contrasting genotypes were selected from the 219 genotypes for morphological, physiological, and biochemical analysis. The selection criteria were based on the consistent performance and host reaction categories observed across the 2-year screening (2016-17 and 2017-2018) (**Figure 2**). Genotypes Varuna, RGN-13, and Pusa Mustard-25 were selected from the MR category; LET-18 and Pusa Mustard-26 from the MS category; EJ-17 and RGN-48 from the S category; and Giriraj, Anuradha, and RGN-13 from the HS category.

### 3.2 Performance analysis of genotypes for morphological and yield aspects

All 10 genotypes were inoculated against spore suspensions of isolated *A. brassicicola* (**Supplementary Figure 1**), and different genotypes showed varying responses. In control plots (mancozeb spray) genotypes in the rabi season of 2018-19 showed distinct AUDPC scores compared to the results from a 2-year screening under natural conditions. Analysis of variance showed that treatments had significant effects on all morphological and yield attributes (**Table 2**). However, genotypes Varuna, Kranti, PM 25, Anuradha, EJ-17, RGN 13, and RGN 48 showed no significant difference in AUDPC scores between inoculated and non-inoculated conditions. Notably, Varuna and Kranti consistently had lower AUDPC scores in both conditions. On the other hand, Giriraj, PM 26, and LET 18 had AUDPC scores ranging from 251 to 500 in non-inoculated conditions, but these scores increased beyond 500 after they were exposed to infection in inoculated conditions. The Varuna genotype showed the highest YPP (18.020 g) in non-inoculated conditions, followed by Kranti (15.480 g). Both Varuna and Kranti experienced reductions in terms of percent change over inoculated conditions of approximately 28% and 36%, respectively. Meanwhile, Giriraj showed YPP (13.110 g) in non-inoculated conditions and showed the highest reduction of 43% after inoculation against *A. brassicicola* (**Supplementary Table 2**).

**Table 2:** Combined analysis of variance for yield traits of 10 mustard genotypes under inoculated and non-inoculated conditions.

Source of variation	Df	PH	PB	SB	MRL	SMR	SL	SPS	SPP	TW	YPP	AUDPC
**Genotypes (G)**	9	3897.28^***^	6.15^***^	54.46^***^	828.67^***^	309.78^***^	3.95^***^	19.85^***^	4224.99^***^	0.58^***^	45.70^***^	130714.68^***^
**Treatment (E)**	1	2969.89^***^	7.95^***^	29.95^***^	580.51^***^	476.27^***^	5.35^***^	52.21^***^	4493.50^***^	6.09^***^	219.42^***^	73290.2^***^
**Interaction (G X E)**	9	9.16**^ns^**	0.20^*^	0.36**^ns^**	6.93**^ns^**	2.86**^ns^**	0.08^*^	0.65**^ns^**	39.10**^ns^**	0.06**^ns^**	2.45^***^	3335.48^***^
**Error**	38	75.86	0.08	0.23	9.15	4.54	0.04	0.42	87.72	0.05	0.28	657.92
**Total**	59											

PH, Plant Height; PB, primary branch number; SB, secondary branch number; MRL, main raceme length; SMR, siliqua on main raceme number; SL, siliqua length; SPS, seeds per siliqua number; SPP, siliqua per plant number; TW, test weight; YPP, seed yield per plant; AUDPC, area under disease progress curve.

(* significant at P<0.05, and *** significant at P<0.001).

### 3.3 Exploring trait associations in inoculated and non-inoculated conditions

**Figure 3:**
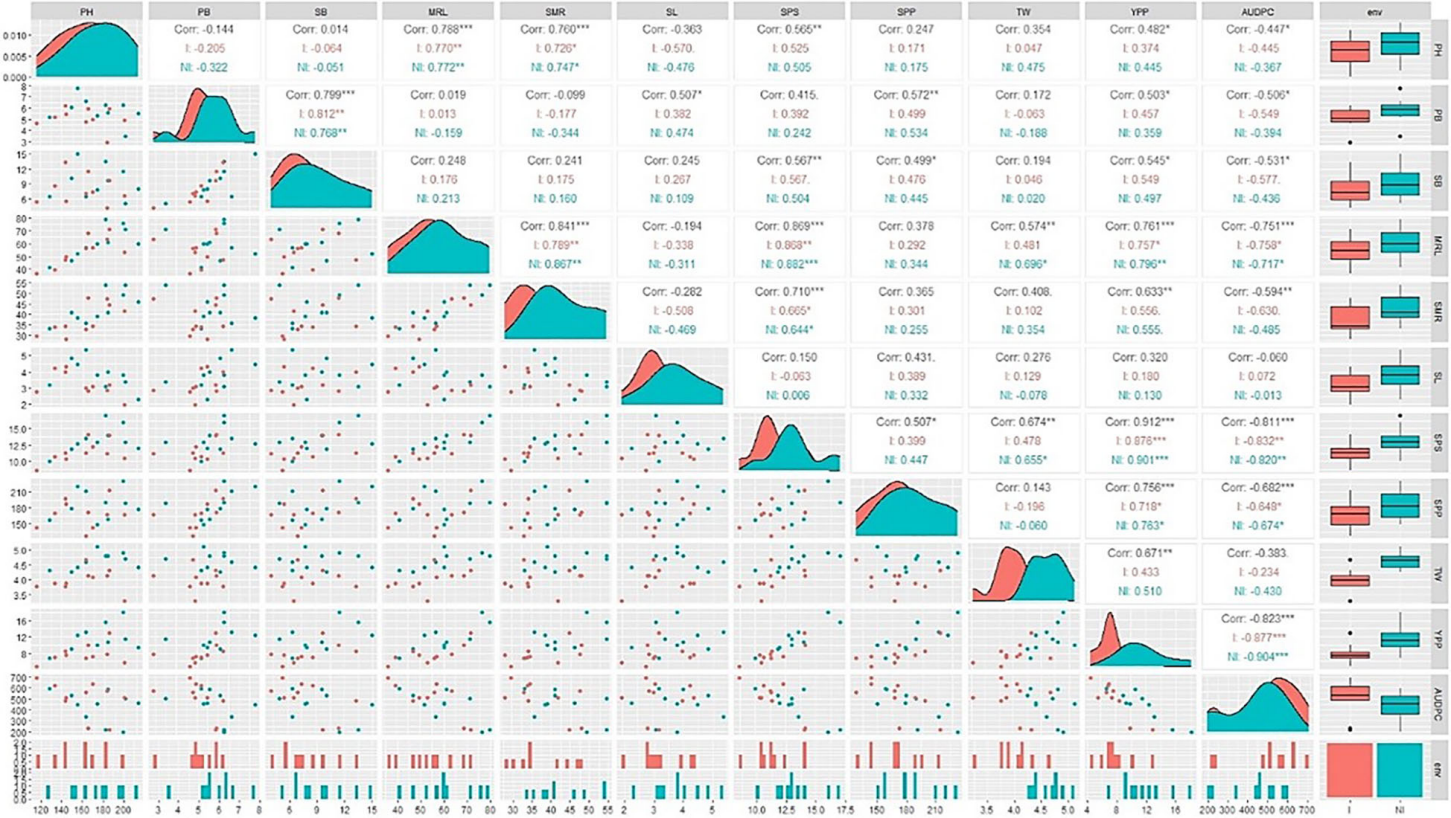
Correlation matrix, scatter plot, and data distribution for yield traits in two conditions, with diagonals indicating the distribution of each parameter and the lower triangular matrix indicating scatter plot. Correlation values and their statistical significance are denoted by asterisks and positioned above the diagonal. Red and navy-blue colors represent correlations within the non-inoculated (NI) and inoculated (I) conditions, respectively. Significance levels are as follows: *** for p ≤ 0.001, ** for p ≤ 0.01, and * for p ≤ 0.05. PH, Plant Height; PB, Primary Branch Number; SB, Secondary Branch Number; MRL, Main Raceme Length; SMR, Siliqua on Main Raceme Number; SL, Siliqua Length; SPS, Seeds per Siliqua Number; SPP, Siliqua per Plant Number; TW, Test Weight; YPP, Seed Yield per Plant; AUDPC, Area Under Disease Progress Curve; Trt, Treatment; NI, Non-Inoculated; I, Inoculated.

For both the inoculated and non-inoculated conditions, area graphs were strategically positioned along the diagonal, and box and whisker plots were situated on the right side of the visual canvas to depict a unified trend. The area graph exhibited a leftward inclination, and the box and whisker plot showed a decrease for all traits following *A. brassicicola* inoculation. It contributed to a cohesive narrative. Notably, the AUDPC, treated as a singular entity, showed a distinct trajectory. In the case of the AUDPC, the area graph demonstrated a rightward inclination, and the box and whisker plot exhibited an increase under inoculated conditions compared to non-inoculated conditions (**Figure 3**).

A correlation coefficient (r) analysis of the traits under both inoculated and non-inoculated conditions was also conducted. The AUDPC demonstrated a robust, negative correlation with MRL, SPS, SPP, and SMR. In contrast, yield had a strong, positive association with these same traits. Notably, the correlation between yield and the AUDPC showed a significant negative correlation (-0.823). It showed an inverse relationship between yield and AUDPC (**Figure 3**).

### 3.4 Principal component analysis

**Figure 4:**
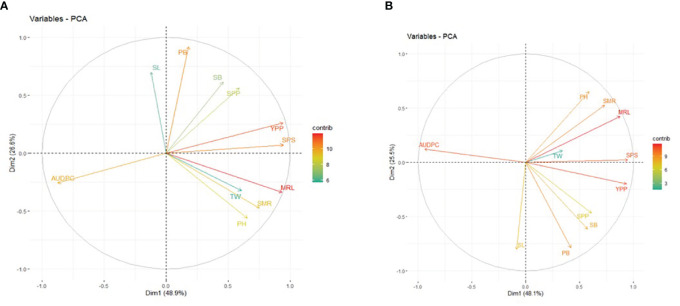
Principal Component Analysis (PCA) Biplot of PC1 and PC2: Contributions of Morphological Traits in **(A)** Non-Inoculated **(B)** Inoculated Conditions. PH, Plant Height; PB, Primary Branch Number; SB, Secondary Branch Number; MRL, Main Raceme Length; SMR, Siliqua on Main Raceme Number; SL, Siliqua Length; SPS, Seeds per Siliqua Number; SPP, Siliqua per Plant Number; TW, Test Weight; YPP, Seed Yield per Plant; AUDPC, Area Under Disease Progress Curve. The contribution to phenotypic variation is represented by the color and lengths of the vector.

The results of the PCA revealed the presence of three principal components (PCs) with eigenvalues exceeding 1 under non-inoculated conditions (5.29, 2.80, and 1.32). The first and second PCs individually explained 48.90% and 26.60% of the phenotypic variance, and the cumulative value was 75.50%. The prominent contributing parameters to these two PCs included MRL, YPP, SPS, PB, AUDPC, SMR, and PH (**Figure 4A**). Conversely, under inoculated conditions, the first three principal components exhibited eigenvalues exceeding or equal to 1 (5.38, 2.93, and 1.00). The first and second PCs independently elucidated approximately 48.10% and 25.50% of the phenotypic variation, collectively amounting to 73.60%. Noteworthy contributors to PC1 and PC2 included MRL, YPP, AUDPC, SPS, SMR, PB, PH, SB, SL, and SPP (**Figure 4B**). It is pertinent to note that under non-inoculated conditions, the AUDPC contributed 9.99% to the overall variability, while its contribution increased to 11.09% under inoculated conditions.

### 3.5 Exploring molecular and biochemical insights

**Figure 5:**
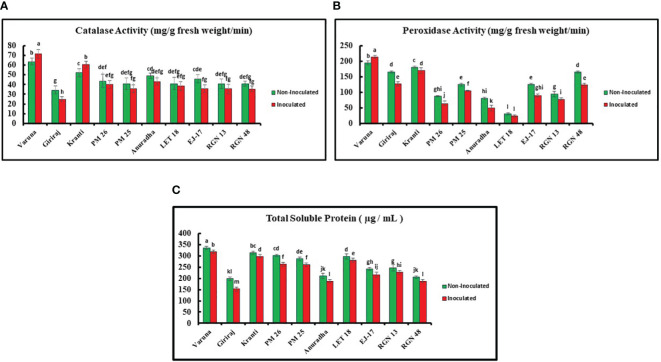
**(A)** Catalase **(B)** Peroxidase Activities **(C)** Total Soluble Protein Content in Mustard Genotypes under Non-Inoculated and Inoculated Conditions. Bars labeled with the same letter indicate no statistically significant difference at a significance level of P<0.05, as determined by the Duncan Multiple Range Test. The average of three determinations is represented by the bars, and error bars indicate the standard error (SE).

The catalase activity was examined for all 10 genotypes and Varuna showed the highest activity (value), while Giriraj exhibited the lowest value under both non-inoculated and inoculated conditions. Specifically, Varuna showed a 13.00% increase in catalase activity under inoculated conditions as compared to the non-inoculated conditions, while Giriraj showed a 27.00% decrease in catalase activity (**Figure 5A**).

Furthermore, Varuna showed significantly higher peroxidase activity than LET-18 in both non-inoculated and inoculated conditions among the tested genotypes, while LET-18 exhibited considerably lower activity. Remarkably, the inoculated Varuna genotype showed a 10.00% increase in peroxidase activity in contrast to the non-inoculated conditions, whereas Anuradha showed a substantial decrease in peroxidase activity (38.00%) (**Figure 5B**).

The TSP content of all 10 genotypes was also estimated. Varuna showed the highest TSP content in both non-inoculated and inoculated environments, while Giriraj showed the lowest values. Comparatively, the Varuna genotype TSP content experienced a 5% increase under the non-inoculated compared to the inoculated condition, whereas Giriraj showed a reduction of 23.00% in TSP under the inoculated condition (**Figure 5C**).

### 3.6 Genotype grouping based on SSI

**Figure 6:**
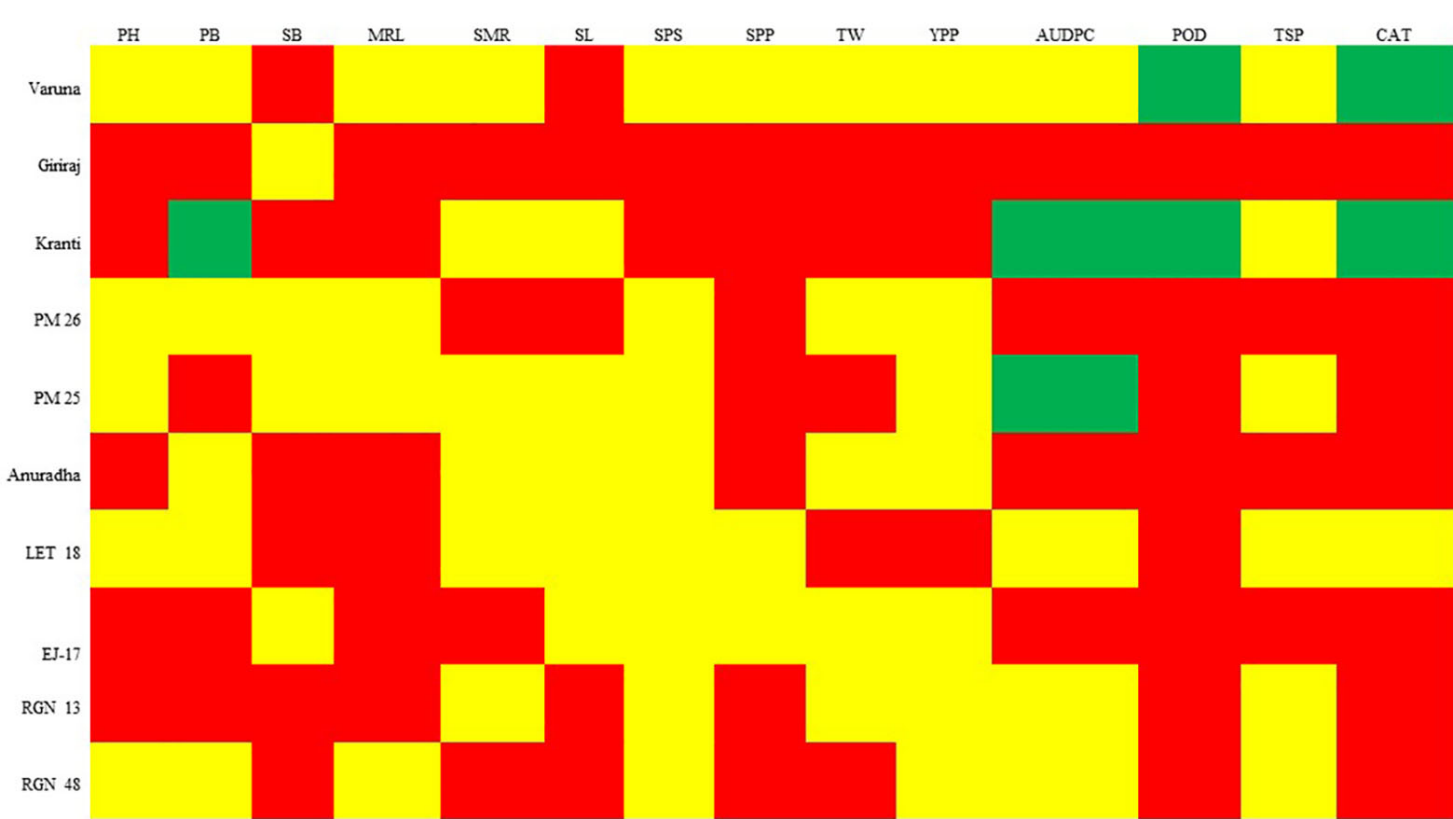
SSI grouping of genotypes based on morpho-physio-biochemical traits. The color green corresponds to an SSI value below 0.5, while the color yellow signifies an SSI value ranging from 0.5 to 1. In contrast, the color red denotes an SSI value exceeding 1. These color codes categorize the genotypes as “resistant,” “moderately resistant,” and “susceptible,” respectively. PH, Plant Height; PB, Primary Branch Number; SB, Secondary Branch Number; MRL, Main Raceme Length; SMR, Siliqua on Main Raceme Number; SL, Siliqua Length; SPS, Seeds per Siliqua Number; SPP, Siliqua per Plant Number; TW, Test Weight; YPP, Seed Yield per Plant; AUDPC, Area Under Disease Progress Curve; POD, Peroxidase Activity; TSP, Total Soluble Protein; CAT, Catalase Activity.

The genotypes were categorized into either “moderately resistant” or “susceptible” groups based on their performance across various morpho-physio-biochemical traits, including PH, SB, MRL, SMR, SL, SPS, SPP, TW, YPP, AUDPC, and TSP (**Figure 6**). Specifically, Genotype Kranti falls into the “resistance” category for traits such as PB, AUDPC, POD, and CAT. Genotype Varuna was categorized as resistant for POD and CAT traits, while PM 25 for AUDPC score. For the remaining traits, these genotypes were categorized as either “moderately resistant” or “susceptible.” Genotype Varuna was predominantly classified as either “resistant” or “moderately resistant” across most traits except SB and SL. In contrast, Genotype Giriraj was primarily labeled as “susceptible” for most of the traits, except for SB. Therefore, for amino acid profiling, Varuna was regarded as a “resistant” genotype, while Giriraj was considered “susceptible.

### 3.7 Amino acid profiling of contrasting mustard genotypes

**Figure 7:**
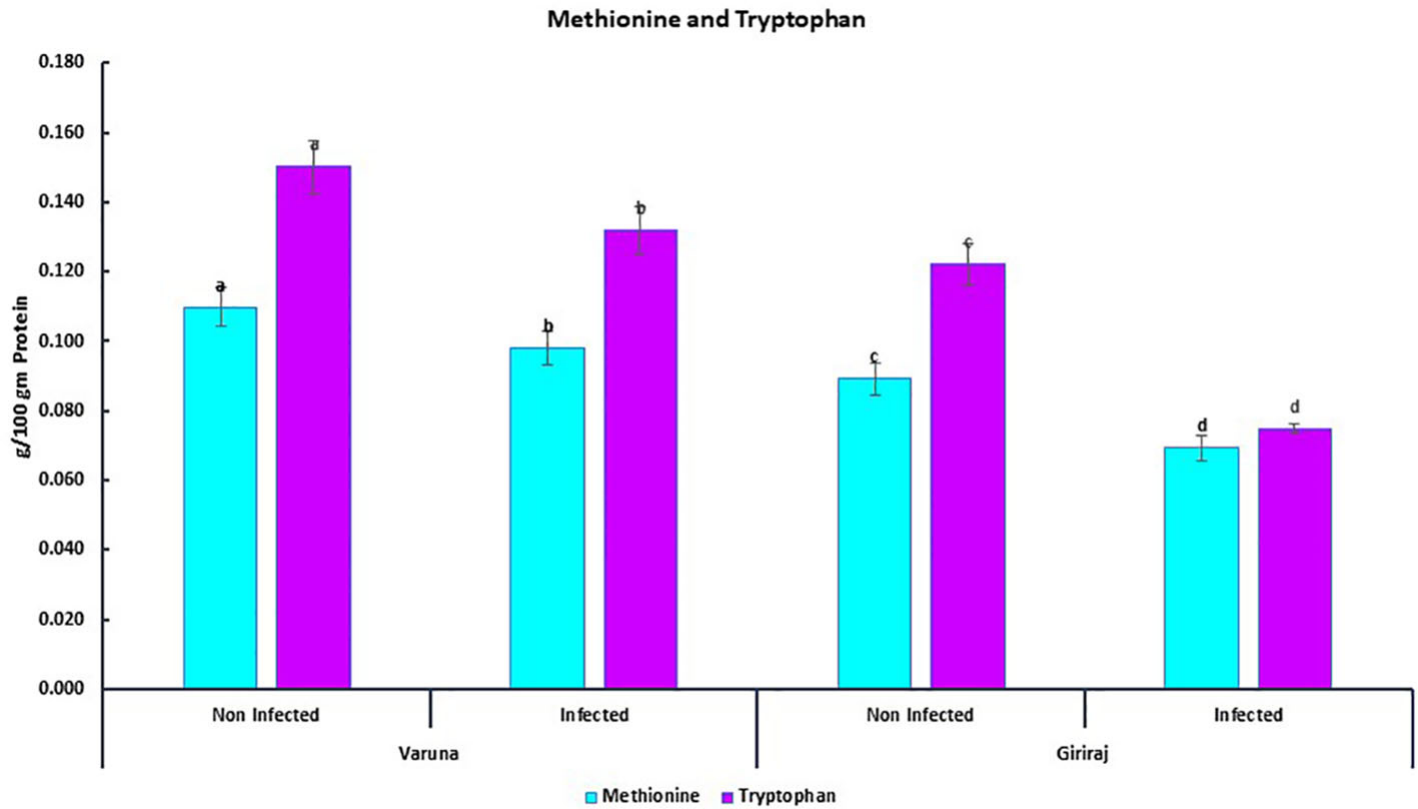
The concentration of methionine and tryptophan in Varuna and Giriraj under non-inoculated and inoculated conditions analyzed using RP-HPLC. Bars labeled with the same letter indicate no statistically significant difference at a significance level of P<0.05, as determined by the Duncan Multiple Range Test. The average of three determinations is represented by the bars, and error bars indicate the standard error (SE).

We also quantified two essential amino acids, methionine and tryptophan, in two contrasting genotypes of mustard (Varuna and Giriraj) seeds. The concentrations of these amino acids were examined under both non-inoculated and inoculated conditions (**Figure 7**). The results revealed that both the Varuna and Giriraj genotypes experienced a decrease in the levels of methionine and tryptophan in the inoculated conditions in comparison to the non-inoculated conditions. Specifically, Varuna displayed an 11% reduction in methionine content, while Giriraj exhibited a more substantial 22% decrease. For tryptophan content, Varuna showed a 12% decrease, while Giriraj demonstrated a notable 39% reduction. Therefore, the level of reduction in amino acids was less for the Alternaria blight-resistant genotype.

## 4 Discussion

Biotic stress factors have been observed to impede plant growth and induce unfavorable alterations at both the cellular and molecular levels (Møller et al., 2007). Despite extensive research endeavors, there has been no substantial discovery of materials that confer a high degree of resistance against *A. brassicicola* (Nowicki et al., 2012; Meena et al., 2020; Singh et al., 2021a). While wild Brassica species have demonstrated considerable resistance (Dharmendra et al., 2014), their compatibility with susceptible cultivated varieties remains limited (Nowakowska et al., 2019). Only very limited resistance has been identified in cultivated Brassica species. Consequently, the identification of resistant genotypes within cultivated species assumes pivotal importance for the success of breeding programs in this context.

To facilitate this objective, effective assessment tools for evaluating pathogen resistance are of paramount significance, and it is prudent to subject germplasm to examination during genuine epidemic occurrences. Building on this perspective, previous studies conducted by Summuna et al. (2012), and Singh et al. (2021a) categorized plant varieties into five distinct classes using a modified rating scale introduced by AICRP-RM-2011 (All India Coordinated Research Project - Rapeseed Mustard). Likewise, in our research efforts, we conducted an evaluation of Indian mustard genotypes for their resistance to Alternaria blight over the course of two consecutive growing seasons, specifically in 2016–17 and 2017–18. Within our investigation, we observed that none of the genotypes exhibited resistance to *Alternaria brassicicola*. Instead, they were categorized into four alternative classes based on their reactions to the prevailing field conditions: moderately resistant, moderately sensitive, susceptible, and highly sensitive.

For the performance of rapeseed-mustard genotypes under controlled conditions concerning *Alternaria brassicicola* infection, we selectively identified genotypes from each host reaction group that consistently showed stability over a two-year duration and implemented artificial inoculation during the reproductive phase. A similar study was conducted by Munir et al. (2020), although their focus was on different Brassicaceae species, such as *B. rapa* and *B. napus*. These investigations delved into various plant characteristics, offering valuable insights into plant-pathogen interactions (Saed-Moucheshi et al., 2013).

In the present study, the correlation plot revealed that the AUDPC was inversely correlated with MRL, SPS, SPP, and SMR, while crop yield demonstrated a positive correlation with these growth attributes. This underscores the crucial role of these growth parameters in enhancing plant yield, particularly in the context of disease resistance, and a positive association between growth parameters such as PH, PB, and MRL and plant yield was identified (Poli et al., 2018; Singh et al., 2021a). While siliqua length exhibited a strong negative correlation with the AUDPC, it did not demonstrate significant associations with yield per plant. These results imply that conventional proxies such as siliqua length may not serve as reliable indicators for breeding and selection purposes due to their inverse relationships with disease severity (Naznin et al., 2015; Ali et al., 2018). In contrast, yield displayed a positive correlation with seeds per pod and thousand seed weight. Interestingly, dark leaf spots caused an adverse effect on the formation of healthy seeds within infected Brassica pods. Consequently, severely disease-spotted pods prematurely dried, contracted, and broke open, leading to the premature shedding of shrunken seeds, resulting in yield loss (Allen et al., 1971; Munir et al., 2020). These findings show the importance of prioritizing strategies aimed at enhancing plant survival and facilitating the development of pods with healthier and more abundant seeds. This suggests that it may be more beneficial to focus efforts on Brassica breeding strategies that prioritize these factors rather than solely emphasizing siliqua length (Bennett et al., 2017).

Plants have developed various defense mechanisms to combat invading pathogens. These mechanisms encompass processes such as callose deposition, lignin formation, the production of phytoalexins, generation of reactive oxygen species, induction of pathogenesis-related (PR) proteins, and the presence of enzymes such as peroxidase and catalase (Torres et al., 2006; Almagro et al., 2009; Doughari, 2015; Yadav et al., 2020). Moreover, catalase and peroxidase play pivotal roles in managing excessive H_2_O_2_ production, which is integral to the plant defense response (Hameed et al., 2008, 2009). Some evidence supports the protective function of POD activity in the context of disease resistance against Alternaria (Tyagi et al., 1998; Hameed et al., 2010). In our investigation, peroxidase activity was elevated after pathogen inoculation Alternaria in Varuna and Kranti. Notably, resistant genotypes (Varuna) displayed higher POD activity under inoculated over non-inoculated conditions. It was observed that increased POD activity in mustard genotypes confronting Alternaria blight (Pandey et al., 2018). Our study showed an increase in CAT activity in both Varuna and Kranti. This surge in CAT activity during the disease period implies the scavanging of excess H_2_O_2_ quickly generated within the plants (Mittler, 2002). Significantly, our results highlight that CAT activity was more pronounced in resistant genotypes compared to susceptible ones, emphasizing the crucial role of CAT in the resistance mechanism (Debona et al., 2012; Meena et al., 2016a).

Our research findings showed a significant association between the progression of the disease and the levels of TSP in mustard genotypes. It was proposed that the proliferation of pathogens triggers the synthesis of various enzymatic proteins and can alter the nutritional composition of the substrate, ultimately leading to an increase in its protein content (Onifade and Jeff-Agboola, 2003). Amino acids play a pivotal role as substrates in host-pathogen interactions (Titarenko et al., 1993), potentially influencing metabolic processes related to disease resistance and exerting fungistatic effects (Misra et al., 2008; Mathpal et al., 2016). These insights shed light on the intricate relationship between disease progression, protein levels, and the role of amino acids in the interactions between plants and pathogens (Moormann et al., 2022).

It is well understood that under stressful conditions, plants engage in photorespiration as a protective mechanism. This process aids in removing light-induced harmful molecules and maintaining the redox balance. It has been hypothesized that photorespiration in plants contributes to the synthesis of sulfur-containing amino acids, including cysteine, and methionine (Kalwan et al., 2023). In mustard crops, growth regulators derived from methionine and tryptophan operate both independently and collaboratively, contributing to plant resistance to biotic stress (Zemanová et al., 2014). Meanwhile, within the Brassicaceae plant family, secondary metabolites known as glucosinolates, which originate from the sulfur-containing amino acid methionine (aliphatic glucosinolates) and tryptophan (indole glucosinolates), play a vital role in enhancing plant immunity and serve as an inducible defense mechanism against pathogens (Choudhury et al., 2022). Notably, our study revealed significant differences in the levels of methionine and tryptophan between the Varuna and Giriraj genotypes with Varuna showing higher levels of these amino acids. These differences suggest a potential role for methionine and tryptophan in enhancing resistance in mustard seeds. These findings raise questions about the relationship between seed protein content and resistance in various contexts. Interestingly, genotypes with higher protein content demonstrated a notable increase in biotic stress-responsive amino acids, such as methionine and tryptophan, suggesting more resistance to diseases in these high-protein genotypes.

## 5 Conclusion

Comprehensive screening of mustard genotypes suggested that gene pools had moderately resistant genotypes. Further, investigation suggested that the Varuna genotype showed the highest resistance compared to the rest of the genotypes against Alternaria blight infection while Giriraj showed the least resistance. Catalase activity increased after infection with the pathogen and the tolerant genotype showed more catalase activity. The observed differences in amino acid content between these genotypes may be linked to the presence of specific resistance genes. Further amino acid profiling is required in more genotypes to confirm its relation with biotic stress tolerance. Moderately tolerant genotypes Varuna and Kranti can be utilized for future experiments and can serve as tolerant material for Alternaria disease. However, further experiments are necessary to precisely identify the resistance genes responsible for modulating tryptophan and methionine content, evaluate their expression, determine their cellular localization, and assess their impact on amino acid levels and protein content in mustard seeds. Identifying these resistance genes would significantly aid in categorizing genotypes based on the relationship between the expression levels of these specific resistance genes and amino acid (methionine and tryptophan) content.

## Data Availability

The original contributions presented in the study are included in the article/**Supplementary Material**. Further inquiries can be directed to the corresponding author.
