# Supplementary material for: Study of resistance mechanism of Alternaria blight (Alternaria brassicicola) by biochemical markers in Indian Mustard (Brassica juncea L. Czern. &Coss.)
Source: Front Plant Sci. 2024 Aug 15;15:1420197. doi: 10.3389/fpls.2024.1420197 (PMC11357941; doi:10.3389/fpls.2024.1420197)
Supplement: Supplementary file 1 [file DataSheet1.docx]

**Supplementary Table 1: Host reaction category and AUDPC scores of selected genotypes in both the years (*rabi* season, 2016-17 and 2017-18) under natural conditions**

| **S. No.** | **Name of genotypes** | **AUDPC** | |  |  | **Host Reaction** | |
| --- | --- | --- | --- | --- | --- | --- | --- |
|  |  | **2016-17** | **2017-18** | **Grade 2016-17** | **Grade 2017-18** | **2016-17** | **2017-18** |
| **1** | TM-277 | 627.20 | 628.45 | 9 | 9 | HS | HS |
| **2** | Rohini | 592.80 | 561.97 | 9 | 9 | HS | HS |
| **3** | NDRE-8-14-1 | 469.00 | 492.45 | 7 | 7 | S | S |
| **4** | BAUM-08-14 | 469.40 | 470.34 | 7 | 7 | S | S |
| **5** | KMR (E) 17-1 | 510.70 | 484.14 | 9 | 7 | HS | S |
| **6** | KMR (E) 17-2 | 474.40 | 498.12 | 7 | 7 | S | S |
| **7** | RLC-6 | 568.10 | 569.24 | 9 | 9 | HS | HS |
| **8** | Pusa Mustard-25 | 95.74 | 94.27 | 3 | 3 | MR | MR |
| **9** | Pusa Mustard-26 | 232.18 | 221.40 | 5 | 5 | MS | MS |
| **10** | RH-1590 | 543.40 | 544.49 | 9 | 9 | HS | HS |
|  | Check | 545.20 | 538.33 | 9 | 9 | HS | HS |
| **11** | DRMRCI-85 | 582.30 | 552.02 | 9 | 9 | HS | HS |
| **12** | RH-1607 | 598.00 | 627.90 | 9 | 9 | HS | HS |
| **13** | RH-1699-22 | 536.20 | 537.27 | 9 | 9 | HS | HS |
| **14** | DRMRIJ-16-66 | 572.40 | 542.64 | 9 | 9 | HS | HS |
| **15** | Anuradha | 633.60 | 665.28 | 9 | 9 | HS | HS |
| **16** | JM-12-6 | 560.50 | 561.62 | 9 | 9 | HS | HS |
| **17** | PR-2015-1 | 519.80 | 492.77 | 9 | 7 | HS | S |
| **18** | CS-2005-137 | 434.90 | 456.65 | 7 | 7 | S | S |
| **19** | CS-2009-129 | 574.10 | 544.25 | 9 | 9 | HS | HS |
| **20** | LET 18 | 218.47 | 217.34 | 5 | 5 | MS | MS |
|  | Check | 548.41 | 551.38 | 9 | 9 | HS | HS |
| **21** | DRMRCI-106 | 563.60 | 534.29 | 9 | 9 | HS | HS |
| **22** | DRMRCI-98 | 514.30 | 540.02 | 9 | 9 | HS | HS |
| **23** | DRMR-2017-14 | 553.10 | 524.34 | 9 | 9 | HS | HS |
| **24** | DRMRIJ-16-38 | 531.90 | 532.96 | 9 | 9 | HS | HS |
| **25** | ET-17 | 439.00 | 441.00 | 7 | 7 | S | S |
| **26** | SKM-1328 | 482.50 | 506.63 | 7 | 9 | S | HS |
| **27** | PR-2015-5 | 512.40 | 485.76 | 9 | 7 | HS | S |
| **28** | SVJ-111 | 451.50 | 452.40 | 7 | 7 | S | S |
| **29** | RB-94 | 530.10 | 502.53 | 9 | 9 | HS | HS |
| **30** | RH-1650 | 471.60 | 495.18 | 7 | 7 | S | S |
|  | Check | 542.42 | 537.60 | 9 | 9 | HS | HS |
| **31** | RH-1656 | 499.50 | 473.53 | 7 | 7 | S | S |
| **32** | PRE-2015-1 | 555.70 | 556.81 | 9 | 9 | HS | HS |
| **33** | PRE-2013-3 | 525.70 | 498.36 | 9 | 7 | HS | S |
| **34** | TM-179 | 461.10 | 462.02 | 7 | 7 | S | S |
| **35** | AKMS-9026 | 623.70 | 654.89 | 9 | 9 | HS | HS |
| **36** | Giriraj | 555.20 | 526.33 | 9 | 9 | HS | HS |
| **37** | NPJ-209 | 562.70 | 563.83 | 9 | 9 | HS | HS |
| **38** | NPJ-210 | 580.90 | 609.95 | 9 | 9 | HS | HS |
| **39** | RGN-435 | 524.40 | 497.13 | 9 | 7 | HS | S |
| **40** | PBR-438 | 450.50 | 451.40 | 7 | 7 | S | S |
|  | Check | 554.43 | 547.77 | 9 | 9 | HS | HS |
| **41** | PBR-400 | 459.20 | 460.12 | 7 | 7 | S | S |
| **42** | PHR-3278 | 584.30 | 553.92 | 9 | 9 | HS | HS |
| **43** | RH-1550 | 528.10 | 554.51 | 9 | 9 | HS | HS |
| **44** | RGN-13 | 580.11 | 586.40 | 9 | 9 | HS | HS |
| **45** | KMR-17-4 | 438.40 | 415.60 | 7 | 7 | S | S |
| **46** | NPJ-211 | 681.10 | 715.16 | 9 | 9 | HS | HS |
| **47** | DRMR-2017-5 | 545.90 | 546.99 | 9 | 9 | HS | HS |
| **48** | NPJ-213 | 604.40 | 572.97 | 9 | 9 | HS | HS |
| **49** | NPJ-214 | 557.90 | 585.80 | 9 | 9 | HS | HS |
| **50** | DRMRCI-91 | 479.80 | 480.76 | 7 | 7 | S | S |
|  | Check | 562.32 | 544.87 | 9 | 9 | HS | HS |
| **51** | DRMR-2017-15 | 446.30 | 423.09 | 7 | 7 | S | S |
| **52** | RH-1569 | 454.60 | 477.33 | 7 | 7 | S | S |
| **53** | LES-56 | 529.60 | 530.66 | 9 | 9 | HS | HS |
| **54** | LES-57 | 470.60 | 446.13 | 7 | 7 | S | S |
| **55** | PDZ-9 | 554.30 | 582.02 | 9 | 9 | HS | HS |
| **56** | TKM-17-2 | 521.90 | 522.94 | 9 | 9 | HS | HS |
| **57** | PMH-8 | 567.20 | 537.71 | 9 | 9 | HS | HS |
| **58** | TH-1603 | 640.20 | 672.21 | 9 | 9 | HS | HS |
| **59** | PT-2015-11 | 671.30 | 636.39 | 9 | 9 | HS | HS |
| **60** | NPJ-215 | 449.80 | 450.70 | 7 | 7 | S | S |
|  | Check | 543.62 | 554.44 | 9 | 9 | HS | HS |
| **61** | NPJ-216 | 476.60 | 451.82 | 7 | 7 | S | S |
| **62** | PRL-2013-15 | 467.20 | 490.56 | 7 | 7 | S | S |
| **63** | RGN-48 | 470.60 | 471.54 | 7 | 7 | S | S |
| **64** | RMWR-09-2 | 525.70 | 526.75 | 9 | 9 | HS | HS |
| **65** | RMWR-09-1 | 496.00 | 470.21 | 7 | 7 | S | S |
| **66** | PT-2015-3 | 673.50 | 707.18 | 9 | 9 | HS | HS |
| **67** | RMT-10-13 | 639.90 | 606.63 | 9 | 9 | HS | HS |
| **68** | PDZ-10 | 567.20 | 568.33 | 9 | 9 | HS | HS |
| **69** | RLC-7 | 462.70 | 438.64 | 7 | 7 | S | S |
| **70** | DRMRQ-4 | 474.80 | 498.54 | 7 | 7 | S | S |
|  | Check | 551.14 | 546.63 | 9 | 9 | HS | HS |
| **71** | PRL-2013-17 | 575.70 | 545.76 | 9 | 9 | HS | HS |
| **72** | RGN-419 | 569.70 | 570.84 | 9 | 9 | HS | HS |
| **73** | RAUDT-14-09 | 658.50 | 624.26 | 9 | 9 | HS | HS |
| **74** | RAUDT-14-04 | 617.70 | 618.94 | 9 | 9 | HS | HS |
| **75** | 71J-0001 | 553.50 | 581.18 | 9 | 9 | HS | HS |
| **76** | 71J-0002 | 563.30 | 534.01 | 9 | 9 | HS | HS |
| **77** | TKM-17-1 | 677.60 | 678.96 | 9 | 9 | HS | HS |
| **78** | BAUT-08-01 | 636.20 | 668.01 | 9 | 9 | HS | HS |
| **79** | DRMRCI-72 | 575.30 | 545.38 | 9 | 9 | HS | HS |
| **80** | DRMRCI-92 | 631.10 | 632.36 | 9 | 9 | HS | HS |
|  | Check | 556.18 | 551.22 | 9 | 9 | HS | HS |
| **81** | AMR(L)-17-5 | 568.10 | 569.24 | 9 | 9 | HS | HS |
| **82** | AMR (L)-17-6 | 541.80 | 513.63 | 9 | 9 | HS | HS |
| **83** | RGN-431 | 459.70 | 482.69 | 7 | 7 | S | S |
| **84** | RH-1518 | 482.00 | 482.96 | 7 | 7 | S | S |
| **85** | RH-1555 | 580.00 | 549.84 | 9 | 9 | HS | HS |
| **86** | BAUM-09-13-1 | 598.80 | 628.74 | 9 | 9 | HS | HS |
| **87** | TM-143 | 545.80 | 546.89 | 9 | 9 | HS | HS |
| **88** | RRN-935 | 627.30 | 594.68 | 9 | 9 | HS | HS |
| **89** | RB-76 | 585.40 | 614.67 | 9 | 9 | HS | HS |
| **90** | TN-117 | 542.20 | 543.28 | 9 | 9 | HS | HS |
|  | Check | 546.20 | 543.44 | 9 | 9 | HS | HS |
| **91** | HUJM-16-8 | 549.00 | 520.45 | 9 | 9 | HS | HS |
| **92** | AKMS-8138 | 563.90 | 592.10 | 9 | 9 | HS | HS |
| **93** | KM-927 | 511.60 | 512.62 | 9 | 9 | HS | HS |
| **94** | YSKM-17-2 | 695.80 | 659.62 | 9 | 9 | HS | HS |
| **95** | EYS-2015-03 | 632.50 | 664.13 | 9 | 9 | HS | HS |
| **96** | EYS-2015-04 | 652.20 | 653.50 | 9 | 9 | HS | HS |
| **97** | RAUDYS-14-05 | 690.70 | 654.78 | 9 | 9 | HS | HS |
| **98** | TMEPAU-103 | 553.10 | 580.76 | 9 | 9 | HS | HS |
| **99** | RTM-314 | 550.40 | 521.78 | 9 | 9 | HS | HS |
| **100** | Kranti | 92.00 | 90.00 | 3 | 3 | MR | MR |
|  | Check | 546.40 | 553.78 | 9 | 9 | HS | HS |
| **101** | RL-1359 | 576.70 | 546.71 | 9 | 9 | HS | HS |
| **102** | NRCYS-05-02 | 705.50 | 740.78 | 9 | 9 | HS | HS |
| **103** | PDZ-1 | 560.50 | 531.35 | 9 | 9 | HS | HS |
| **104** | RHH-1687 | 602.10 | 603.30 | 9 | 9 | HS | HS |
| **105** | RRN-917 | 528.90 | 501.40 | 9 | 9 | HS | HS |
| **106** | KGS-35 | 562.20 | 590.31 | 9 | 9 | HS | HS |
| **107** | PHR-1500 | 523.30 | 496.09 | 9 | 7 | HS | S |
| **108** | SVJH-100 | 580.40 | 581.56 | 9 | 9 | HS | HS |
| **109** | Pusa MH-8 | 548.20 | 519.69 | 9 | 9 | HS | HS |
| **110** | RAUTYS-14-09 | 740.30 | 777.32 | 9 | 9 | HS | HS |
|  | Check | 561.62 | 557.22 | 9 | 9 | HS | HS |
| **111** | NDYS-424 | 675.10 | 639.99 | 9 | 9 | HS | HS |
| **112** | YSH-0401(NC) | 683.20 | 684.57 | 9 | 9 | HS | HS |
| **113** | AKMS-8141 | 572.50 | 542.73 | 9 | 9 | HS | HS |
| **114** | Pusa MH-9 | 550.50 | 551.60 | 9 | 9 | HS | HS |
| **115** | Pitambari | 683.30 | 717.47 | 9 | 9 | HS | HS |
| **116** | BIOYSR | 521.90 | 494.76 | 9 | 7 | HS | S |
| **117** | 71J-0004 | 552.10 | 553.20 | 9 | 9 | HS | HS |
| **118** | YSKM-17-1 | 669.20 | 702.66 | 9 | 9 | HS | HS |
| **119** | PT-303 | 714.00 | 676.87 | 9 | 9 | HS | HS |
| **120** | GSL-1 | 87.80 | 87.98 | 3 | 3 | MR | MR |
|  | Check | 551.52 | 562.53 | 9 | 9 | HS | HS |
| **121** | DLSC-1 | 85.20 | 85.37 | 3 | 3 | MR | MR |
| **122** | DRMRIC-16-38 | 552.50 | 523.77 | 9 | 9 | HS | HS |
| **123** | DRMRHJ-2503 | 103.90 | 109.10 | 5 | 5 | MS | MS |
| **124** | CS-13000-3-1-1-4-2 | 514.00 | 515.03 | 9 | 9 | HS | HS |
| **125** | CS-2009-154 | 513.00 | 486.32 | 9 | 7 | HS | S |
| **126** | CS-2009-335 | 536.30 | 563.12 | 9 | 9 | HS | HS |
| **127** | CS-15000-1-1-1-1-2 | 496.80 | 497.79 | 7 | 7 | S | S |
| **128** | DRMR-4005 | 522.40 | 495.24 | 9 | 7 | HS | S |
| **129** | SVJ-68 | 549.80 | 577.29 | 9 | 9 | HS | HS |
| **130** | RH-1573 | 593.40 | 594.59 | 9 | 9 | HS | HS |
|  | Check | 548.36 | 543.33 | 9 | 9 | HS | HS |
| **131** | PRE-2013-1 | 635.90 | 602.83 | 9 | 9 | HS | HS |
| **132** | RH-1326 | 566.20 | 594.51 | 9 | 9 | HS | HS |
| **133** | RH-923 | 564.10 | 565.23 | 9 | 9 | HS | HS |
| **134** | RH-919 | 513.90 | 487.18 | 9 | 7 | HS | S |
| **135** | DRMR-1165-40 | 479.00 | 502.95 | 7 | 9 | S | HS |
| **136** | TS-38 | 670.00 | 671.34 | 9 | 9 | HS | HS |
| **137** | NPJ-203 | 609.70 | 578.00 | 9 | 9 | HS | HS |
| **138** | RGN-761 | 636.00 | 667.80 | 9 | 9 | HS | HS |
| **139** | TH-1402 | 667.20 | 632.51 | 9 | 9 | HS | HS |
| **140** | RAUDT-10-33 | 663.30 | 664.63 | 9 | 9 | HS | HS |
|  | Check | 557.51 | 546.21 | 9 | 9 | HS | HS |
| **141** | PHR-2 | 195.00 | 184.86 | 5 | 5 | MS | MS |
| **142** | RGN-394 | 545.20 | 572.46 | 9 | 9 | HS | HS |
| **143** | RH-1209 | 491.10 | 465.56 | 7 | 7 | S | S |
| **144** | RLMCP-626 | 567.00 | 568.13 | 9 | 9 | HS | HS |
| **145** | KMR (L)-15-5 | 531.20 | 503.58 | 9 | 9 | HS | HS |
| **146** | PBR-422 | 494.60 | 519.33 | 7 | 9 | S | HS |
| **147** | PRO-5222 | 530.60 | 503.01 | 9 | 9 | HS | HS |
| **148** | DRMR-1153-12 | 537.30 | 538.37 | 9 | 9 | HS | HS |
| **149** | LES-54 | 491.70 | 466.13 | 7 | 7 | S | S |
| **150** | EC-399299 | 424.40 | 445.62 | 7 | 7 | S | S |
|  | Check | 549.38 | 542.72 | 9 | 9 | HS | HS |
| **151** | CS-700-2-1-4 | 525.60 | 498.27 | 9 | 7 | HS | S |
| **152** | CS-900-1-2-2-1-3 | 512.70 | 513.73 | 9 | 9 | HS | HS |
| **153** | CS-508-1-P2 | 547.90 | 519.41 | 9 | 9 | HS | HS |
| **154** | RGN-368 | 555.00 | 556.11 | 9 | 9 | HS | HS |
| **155** | TS-46 | 577.30 | 606.17 | 9 | 9 | HS | HS |
| **156** | DRMR-2035 | 585.90 | 555.43 | 9 | 9 | HS | HS |
| **157** | PT-2010-5 | 603.40 | 604.61 | 9 | 9 | HS | HS |
| **158** | YSB-9 | 681.10 | 715.16 | 9 | 9 | HS | HS |
| **159** | PAB-16-2 | 382.20 | 362.33 | 7 | 7 | S | S |
| **160** | PRD-14-1 | 245.10 | 245.59 | 5 | 5 | MS | MS |
|  | Check | 559.43 | 545.55 | 9 | 9 | HS | HS |
| **161** | NDRS-2008-1 | 539.20 | 511.16 | 9 | 9 | HS | HS |
| **162** | NDRS-2011 | 494.80 | 519.54 | 7 | 9 | S | HS |
| **163** | PAB-14-5 | 208.50 | 208.92 | 5 | 5 | MS | MS |
| **164** | PRD-14-6 | 234.30 | 222.12 | 5 | 5 | MS | MS |
| **165** | DRMRSJ-31 | 291.60 | 306.18 | 7 | 7 | S | S |
| **166** | PRD-14-11 | 205.20 | 205.61 | 5 | 5 | MS | MS |
| **167** | PRD-14-16 | 222.60 | 211.02 | 5 | 5 | MS | MS |
| **168** | PRD-14-18 | 238.80 | 250.74 | 5 | 7 | MS | S |
| **169** | JMM-991 | 236.60 | 237.07 | 5 | 5 | MS | MS |
| **170** | RMM-09-04 | 288.80 | 273.78 | 7 | 7 | S | S |
|  | Check | 552.28 | 550.18 | 9 | 9 | HS | HS |
| **171** | RMM-09-06 | 361.80 | 379.89 | 7 | 7 | S | S |
| **172** | PAB-14-14 | 189.80 | 190.18 | 5 | 5 | MS | MS |
| **173** | PAB-14-17 | 219.20 | 207.80 | 5 | 5 | MS | MS |
| **174** | PBZ-4 | 199.40 | 209.37 | 5 | 5 | MS | MS |
| **175** | PBZ-5 | 257.10 | 257.61 | 7 | 7 | S | S |
| **176** | PBZ-7 | 306.10 | 290.18 | 7 | 7 | S | S |
| **177** | DRMR-1-5 | 465.80 | 489.09 | 7 | 7 | S | S |
| **178** | DRMR-2019 | 436.50 | 437.37 | 7 | 7 | S | S |
| **179** | NDRS-2009-1 | 547.00 | 574.35 | 9 | 9 | HS | HS |
| **180** | PDZ-2 | 512.10 | 485.47 | 9 | 7 | HS | S |
|  | Check | 558.24 | 548.61 | 9 | 9 | HS | HS |
| **181** | PDZ-3 | 540.40 | 567.42 | 9 | 9 | HS | HS |
| **182** | DRMR-2-11 | 487.00 | 487.97 | 7 | 7 | S | S |
| **183** | DRMR-5206 | 534.10 | 560.81 | 9 | 9 | HS | HS |
| **184** | DRMRSJ-9-1 | 584.00 | 553.63 | 9 | 9 | HS | HS |
| **185** | RH-1372 | 498.40 | 523.32 | 7 | 9 | S | HS |
| **186** | RH-1378 | 484.80 | 485.77 | 7 | 7 | S | S |
| **187** | NPJ-217 | 540.80 | 567.84 | 9 | 9 | HS | HS |
| **188** | EC-399301 | 490.60 | 465.09 | 7 | 7 | S | S |
| **189** | NPJ-218 | 519.30 | 545.27 | 9 | 9 | HS | HS |
| **190** | NPJ-219 | 474.50 | 475.45 | 7 | 7 | S | S |
|  | Check | 551.42 | 557.48 | 9 | 9 | HS | HS |
| **191** | NPJ-220 | 508.90 | 534.35 | 9 | 9 | HS | HS |
| **192** | DRMRSJ-9-1-1 | 450.80 | 427.36 | 7 | 7 | S | S |
| **193** | RMT-15-29 | 574.10 | 602.81 | 9 | 9 | HS | HS |
| **194** | RRN-911 | 528.80 | 555.24 | 9 | 9 | HS | HS |
| **195** | DRMRCI-55 | 458.40 | 434.56 | 7 | 7 | S | S |
| **196** | NPJ-201 | 609.90 | 611.12 | 9 | 9 | HS | HS |
| **197** | RB-72 | 473.90 | 497.60 | 7 | 7 | S | S |
| **198** | SVJ-72 | 450.00 | 426.60 | 7 | 7 | S | S |
| **199** | PRD-2013-6 | 521.00 | 522.04 | 9 | 9 | HS | HS |
| **200** | RHH 1561 | 478.10 | 502.01 | 7 | 9 | S | HS |
|  | Check | 559.14 | 543.11 | 9 | 9 | HS | HS |
| **201** | DRMRHJ 913 | 523.50 | 496.28 | 9 | 7 | HS | S |
| **202** | PHR-126 | 441.90 | 464.00 | 7 | 7 | S | S |
| **203** | SKM-1104 | 490.00 | 490.98 | 7 | 7 | S | S |
| **204** | PR-2013-02 | 471.80 | 447.27 | 7 | 7 | S | S |
| **205** | KMR (E)-16-1 | 484.80 | 509.04 | 7 | 9 | S | HS |
| **206** | DRMRCI-58 | 493.50 | 494.49 | 7 | 7 | S | S |
| **207** | T-9 | 683.20 | 647.67 | 9 | 9 | HS | HS |
| **208** | Shital | 93.50 | 98.18 | 3 | 3 | MR | MR |
| **209** | Jhumka | 634.90 | 636.17 | 9 | 9 | HS | HS |
| **210** | Varuna | 89.00 | 87.00 | 3 | 3 | MR | MR |
|  | Check | 553.67 | 550.78 | 9 | 9 | HS | HS |
| **211** | Parvati | 619.80 | 650.79 | 9 | 9 | HS | HS |
| **212** | Vaibhav | 527.70 | 528.76 | 9 | 9 | HS | HS |
| **213** | Ashirvad | 567.90 | 538.37 | 9 | 9 | HS | HS |
| **214** | Maya | 567.30 | 595.67 | 9 | 9 | HS | HS |
| **215** | Vardan | 508.60 | 509.62 | 9 | 9 | HS | HS |
| **216** | GSL-2 | 104.30 | 98.88 | 5 | 3 | MS | MR |
| **217** | GSL-5 | 110.00 | 115.50 | 5 | 5 | MS | MS |
| **218** | PT-30 | 681.40 | 682.76 | 9 | 9 | HS | HS |
| **219** | T-27 | 608.70 | 639.14 | 9 | 9 | HS | HS |
|  | Check | 558.91 | 547.17 | 9 | 9 | HS | HS |

**Supplementary Table 2**. Duncan’s Multiple Range Test (DMRT) for comparison of mean performance of genotypes for various parameters in two treatment conditions

| Treatment | Genotypes | PH | PB | SB | MRL | SMR | SL | SPS | SPP | TW | YPP | AUDPC |
| --- | --- | --- | --- | --- | --- | --- | --- | --- | --- | --- | --- | --- |
| Non Inoculated | Varuna | 197.4±9.41^abcd^ | 6±0.3^bc^ | 12±0.49^c^ | 76.2±3.64^ab^ | 49±2.27^b^ | 3.8±0.16^e^ | 16±0.72^a^ | 230±10.86^a^ | 4.90±0.22^ab^ | 18.02±0.66^a^ | 198±11.23^j^ |
|  | Giriraj | 162.2±7.36^fgh^ | 7±0.28^b^ | 7±0.29^h^ | 56.6±2.52^ef^ | 36±1.44^fg^ | 5.3±0.22^a^ | 14±0.53^bc^ | 211±9.57^bc^ | 4.59±0.2^bcde^ | 13.11±0.38^c^ | 340±26.02^i^ |
|  | Kranti | 181.3±8.4^e^ | 6±0.3^bc^ | 14±0.58^b^ | 79.4±3.48^a^ | 54±2.43^a^ | 3.1±0.14^fgh^ | 17±0.72^a^ | 190±8.75^de^ | 4.80±0.21^abc^ | 15.48±0.51^b^ | 221±12.04^j^ |
|  | PM 26 | 173.6±8.27^ef^ | 5±0.24^fg^ | 8±0.36^ef^ | 60.8±2.86^de^ | 39±1.68^ef^ | 3.8±0.15^e^ | 13±0.58^bcd^ | 149±6.55^i^ | 5.10±0.24^a^ | 9.91±0.35^ef^ | 458±28.47^h^ |
|  | PM 25 | 154.6±7.3^ghi^ | 8±0.32^a^ | 15±0.68^a^ | 52.1±2.4^fgh^ | 38±1.75^ef^ | 4.5±0.2^c^ | 13±0.58^cd^ | 220±10.07^ab^ | 4.41±0.19^cdef^ | 12.37±0.43^cd^ | 451±24.55^h^ |
|  | Anuradha | 128.1±5.94^kl^ | 5±0.24^fg^ | 7±0.28^h^ | 41.3±1.87^i^ | 33±1.52^gh^ | 3.2±0.14^fg^ | 10±0.44^g^ | 158±7.31^hi^ | 4.31±0.19^def^ | 6.82±0.24^j^ | 592±35.31^cd^ |
|  | LET 18 | 211.2±10.12^a^ | 6±0.25^ef^ | 8±0.34^ef^ | 60.2±2.7^de^ | 46±2.13^bc^ | 2.3±0.1^i^ | 12±0.53^de^ | 178±8.55^efg^ | 4.40±0.17^cdef^ | 9.41±0.29^fg^ | 459±25.97^h^ |
|  | EJ-17 | 148.9±6.81^hi^ | 6±0.27^cd^ | 10±0.44^d^ | 47.5±2.03^h^ | 41±1.74^de^ | 4.8±0.22^b^ | 12±0.54^de^ | 178±8.66^efg^ | 4.25±0.2^efg^ | 8.99±0.36^gh^ | 531±31.79^ef^ |
|  | RGN 13 | 199.3±9.3^ab^ | 4±0.15^i^ | 5±0.21^i^ | 71.3±3.22^bc^ | 54±2.42^a^ | 3.4±0.15^f^ | 13±0.57^bcd^ | 189±8.54^de^ | 4.70±0.21^abcd^ | 11.53±0.39^d^ | 506±29.39^fg^ |
|  | RGN 48 | 183.8±8.6^cde^ | 6±0.25^def^ | 10±0.38^d^ | 61.2±2.91^de^ | 41±1.76^de^ | 4.1±0.16^de^ | 14±0.63^b^ | 158±7.32^hi^ | 4.80±0.21^abc^ | 10.65±0.37^e^ | 586±32.45^cd^ |
| Inoculated | Varuna | 184.4±9.41^bcde^ | 6±0.3^cde^ | 10±0.49^d^ | 71.3±3.64^bc^ | 45±2.27^cd^ | 3.2±0.16^fg^ | 14±0.72^b^ | 213±10.86^bc^ | 4.32±0.22^def^ | 12.89±0.66^c^ | 220±11.23^j^ |
|  | Giriraj | 144.3±7.36^ij^ | 5±0.28^ef^ | 6±0.29^i^ | 49.5±2.52^gh^ | 28±1.44^i^ | 4.3±0.22^c^ | 10±0.53^fg^ | 188±9.57^def^ | 3.87±0.2^gh^ | 7.53±0.38^j^ | 510±26.02f^g^ |
|  | Kranti | 164.6±8.4^fgh^ | 6±0.3^cde^ | 11±0.58^c^ | 68.3±3.48^c^ | 48±2.43^bc^ | 2.8±0.14^h^ | 14±0.72^b^ | 171±8.75^fgh^ | 4.12±0.21^fgh^ | 9.96±0.51^ef^ | 236±12.04^j^ |
|  | PM 26 | 162.4±8.27^fgh^ | 5±0.24^gh^ | 7±0.36g^h^ | 56.1±2.86^ef^ | 33±1.68^gh^ | 3.1±0.15^gh^ | 11±0.58^ef^ | 128±6.55^j^ | 4.67±0.24^bcd^ | 6.78±0.35^j^ | 558±28.47^de^ |
|  | PM 25 | 143.7±7.3^ij^ | 6±0.32^bc^ | 13±0.68^b^ | 47.1±2.4^h^ | 34±1.75^g^ | 4.1±0.2^de^ | 11±0.58^ef^ | 197±10.07^cd^ | 3.78±0.19^h^ | 8.48±0.43^hi^ | 481±24.55^gh^ |
|  | Anuradha | 116.3±5.94^l^ | 5±0.24^h^ | 5±0.28^i^ | 36.6±1.87^i^ | 30±1.52h^i^ | 2.8±0.14^h^ | 9±0.44^h^ | 143±7.31^ij^ | 3.78±0.19^h^ | 4.69±0.24^l^ | 692±35.31^a^ |
|  | LET 18 | 198.3±10.12^abc^ | 5±0.25^gh^ | 7±0.34^gh^ | 53.5±2.7^fg^ | 42±2.13^de^ | 2.1±0.1^j^ | 10±0.53^fg^ | 167±8.55^gh^ | 3.28±0.17^i^ | 5.75±0.29^k^ | 509±25.97^fg^ |
|  | EJ-17 | 133.4±6.81^jk^ | 5±0.27^fg^ | 9±0.44^e^ | 39.7±2.03^i^ | 34±1.74^g^ | 4.2±0.22^cd^ | 11±0.54^fg^ | 170±8.66^gh^ | 3.88±0.2^gh^ | 7.04±0.36^j^ | 623±31.79^bc^ |
|  | RGN 13 | 182.3±9.3^de^ | 3±0.15^j^ | 4±0.21^j^ | 63.2±3.22^d^ | 47±2.42^bc^ | 2.9±0.15^gh^ | 11±0.57^ef^ | 167±8.54^gh^ | 4.12±0.21^fgh^ | 7.74±0.39^ij^ | 576±29.39^d^ |
|  | RGN 48 | 168.6±8.6^efg^ | 5±0.25^gh^ | 7±0.38^fg^ | 57.4±2.91^ef^ | 35±1.76^g^ | 3.1±0.16^fgh^ | 12±0.63^de^ | 144±7.32^ij^ | 4.07±0.21^fgh^ | 7.17±0.37^j^ | 636±32.45^b^ |

PH: Plant Height; PB: primary branch number; SB: secondary branch number; MRL: main raceme length; SMR: siliqua on main raceme number; SL: siliqua length; SPS: seeds per siliqua number; SPP: siliqua per plant number; TW: test weight; YPP: seed yield per plant; AUDPC: area under disease progress curve


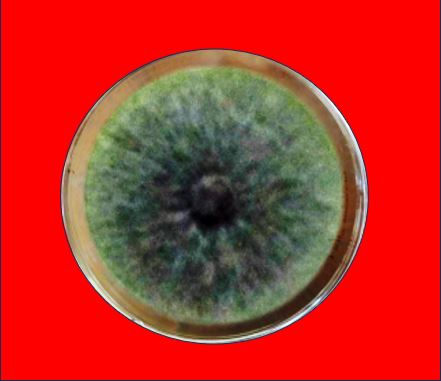

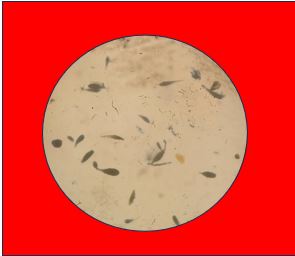


**Inoculated**

**Non-Inoculated**

**A**

**B**

**Supplementary Figure 1 (A)** Culture of *Alternaria brassicicola* isolated from Inoculated Mustard Leaves and Microscopic Image of Conidia **(B)** Alternaria Blight Symptoms on Inoculated Mustard Leaves and Siliqua.
